# Transcriptome Analysis of Liver Cancer Cell Huh-7 Treated With Metformin

**DOI:** 10.3389/fphar.2022.822023

**Published:** 2022-03-23

**Authors:** Chun-Qing Li, Zhi-Qin Liu, Sha-Sha Liu, Gao-Tao Zhang, Li Jiang, Chuan Chen, Du-Qiang Luo

**Affiliations:** ^1^ Key Laboratory of Microbial Diversity Research and Application of Hebei Province, College of Life Science, Hebei University, Baoding, China; ^2^ Key Laboratory of Pharmaceutical Quality Control of Hebei Province, College of Pharmaceutical Science, Hebei University, Baoding, China; ^3^ College of Science and Technology, Hebei Agricultural University, Huanghua, China

**Keywords:** metformin, transcriptome analysis, hepatocellular carcinoma, signaling pathway, Huh-7

## Abstract

Metformin is a kind of widely used antidiabetic drug that regulates glucose homeostasis by inhibiting liver glucose production and increasing muscle glucose uptake. Recently, some studies showed that metformin exhibits anticancer properties in a variety of cancers. Although several antitumor mechanisms have been proposed for metformin action, its mode of action in human liver cancer remains not elucidated. In our study, we investigated the underlying molecular mechanisms of metformin's antitumor effect on Huh-7 cells of hepatocellular carcinoma (HCC) *in vitro*. RNA sequencing was performed to explore the effect of metformin on the transcriptome of Huh-7 cells. The results revealed that 4,518 genes (with log2 fold change > 1 or < −1, adjusted p-value < 0.05) were differentially expressed in Huh-7 cells with treatment of 25-mM metformin compared with 0-mM metformin, including 1,812 upregulated and 2,706 downregulated genes. Gene ontology and Kyoto Encyclopedia of Genes and Genomes pathway analyses identified 54 classical pathways that were significantly enriched, and 16 pathways are closely associated with cancer, such as cell cycle, DNA replication, extracellular matrix–receptor interaction, and so on. We selected 11 differentially expressed genes, which are closely associated with HCC, to validate their differential expressions through a quantitative real-time reverse transcription-polymerase chain reaction. The result exhibited that the genes of fatty acid synthase, mini-chromosome maintenance complex components 6 and 5, myristoylated alanine-rich C-kinase substrate, fatty acid desaturase 2, C-X-C motif chemokine ligand 1, bone morphogenetic protein 4, S-phase kinase-associated protein 2, kininogen 1, and proliferating cell nuclear antigen were downregulated, and Dual-specificity phosphatase-1 is significantly upregulated in Huh-7 cells with treatment of 25-mM metformin. These differentially expressed genes and pathways might play a crucial part in the antitumor effect of metformin and might be potential targets of metformin treating HCC. Further investigations are required to evaluate the metformin mechanisms of anticancer action *in vivo*.

## Introduction

Liver cancer is the most common malignant tumor and the third leading cause of cancer-related mortality worldwide, just next to lung and colorectal cancers. It is estimated that there are approximately 850,000 new cases of liver cancer each year, with a mortality rate of 8.2% of all cancers worldwide (Llovet et al., 2016; Bray et al., 2018). Liver cancer occurs in association with chronic liver injury and hepatocellular death caused by viral hepatitis, chronic alcohol abuse, drugs or chemical insults, obesity, and aflatoxin-B1-contaminated food (Farazi and DePinho, 2006; El-Serag and Rudolph, 2007). Hepatocellular carcinoma (HCC) is the most prevalent type of liver cancer, representing over 80% of all liver cancer cases (Yi et al., 2018). As a highly metastatic and recurrent cancer, HCC tends to be limited with the treatment choices of chemotherapy, surgical resection, liver transplantation, and local ablation in its early stages. Sorafenib is a multikinase inhibitor and has a poor outcome despite being a widely used agent for advanced HCC (Llovet et al., 2008). Similarly, low therapeutic effects were reported for cabozantinib, lenvatinib, and regorafenib (Bruix et al., 2017; Abou-Alfa et al., 2018; Kudo et al., 2018). Therefore, it is urgent to develop new effective drugs to target the HCC cells.

Metformin, as an oral hypoglycemic agent, has been used as the first-line treatment of type 2 diabetes mellitus with a safety profile. Recently, it has been reported that metformin can prevent and control the development of a variety of cancers (Evans et al., 2005; Morales and Morris, 2015).

Abundant epidemiological data have indicated that metformin used in patients with type 2 diabetes mellitus can significantly reduce the incidence of HCC (Lee et al., 2011; Nkontchou et al., 2011; Lee et al., 2012). Moreover, other recent epidemiological studies have demonstrated that metformin treatment can reduce tumor recurrence or improve the prognosis of patients who underwent hepatic resection for HCC (Donadon et al., 2010; Li et al., 2009; Lee et al., 2012; Eisenreich and Leppert, 2017). Several laboratory studies have also confirmed that metformin can inhibit the growth of liver cancer cells by inducing cell arrest and enhancing apoptosis (Buzzai et al., 2007; Ben et al., 2011). Although several mechanisms have been proposed for metformin action, the explicit molecular basis of its antitumor activity in HCC remained incompletely understood. In recent years, with the rapid development of next-generation sequencing technologies, RNA sequencing technology has developed into an effective tool to identify functional genes after treatment with various drugs (Sun et al., 2015). Recently, numerous studies were carried out on the transcriptome profiling of tumor cells for functional gene identification. To gain an intensive insight into the molecular mechanism of metformin treatment on HCC cell Huh-7, we performed a transcriptome screening analysis to identify gene targets and signaling pathways through which metformin might have influenced HCC. Meanwhile, this research is expected to provide an experimental basis for the application of metformin in HCC treatment and help to suggest directions for future investigation.

## Materials and Methods

### Chemicals and Reagents

Dulbecco's modified Eagle's medium (DMEM, 1 g/L D-glucose) was purchased from Gibco. The TRIzol reagent and 0.25% trypsin– ethylenediaminetetraacetic acid (1×) were purchased from Invitrogen (Carlsbad, CA, United States). Fetal bovine serum (900-108) was from GEMINI. 3-(4,5-Dimethylthiazol-2-yl)-2,5-diphenyltetrazolium bromide (MTT, M8180) was obtained from Solarbio (Beijing, China). 1,1-Dimethylbiguanide hydrochloride (Metformin, D150959) was purchased from Sigma-Aldrich (Saint Louis, United States). SYBR Green polymerase chain reaction (PCR) Master Mix was purchased from Vazyme (Nanjing, China). TransScript All-in-One First-Strand complementary DNA (cDNA) Synthesis SuperMix was purchased from TRANSGEN BIOTECH (Beijing, China).

### Cell Lines and Cell Culture

HCC cell line Huh-7 was purchased from the Type Culture Collection of the Chinese Academy of Sciences (Shanghai, China) and cultured in DMEM medium supplemented with 10% fetal bovine serum at 37°C in a culture incubator with 5% CO_2_. The cell culture medium was refreshed every other day. All cell culture experiments were carried out within 10 passages after being thawed.

### 3-(4,5-Dimethylthiazol-2-yl)-2,5-Diphenyltetrazolium Bromide Assay

The exponentially growing Huh-7 cells were grown in 96-well plates with 5,000 cells per well with a 100-µl medium for 24 h and then starved for 12 h at 37°C in a humidified atmosphere containing 5% CO_2_. Subsequently, the Huh-7 cells were treated with the concentrations of 0- and 20-mM metformin dissolved in a complete DMEM. After culturing for 24, 48, and 72 h in a 37°C humidified incubator, 20 µl of 5 mg/ml MTT reagent was added to each well, and the plates were incubated for 4 h. A total of 100 µl of 0.01-M SDS-HCl was then added into each plate and incubated at 37°C overnight, and the optical density value at 570 nm was examined using a microplate reader (Thermo Fisher Scientifc, Inc., Waltham, MA, United States).

### Real-Time Cell Analysis of Metformin

Huh-7 cells in the logarithmic growth phase were planted in 16-well plates (E-plate 16 ACEA Biosciences Inc., San Diego, United States) according to the xCELLigence Real-Time Cell Analyzer (RTCA) DP instrument manual as provided by the manufacturer. To be specific, Huh-7cells were harvested by trypsinization using 0.25% trypsin–ethylenediaminetetraacetic acid and then planted into 16-well plates with 5,000 cells per well with a 100-µl medium. Before planting, cell concentration, aggregation rate, and viability were evaluated by automated Trypan blue exclusion using the Cedex XS Analyzer (Roche, Cedex), and background impedance was measured in 50-μl cell culture medium per well. After planting, impedance was detected in the following schedule: 1-min intervals for 2 h, 5-min intervals for 2 h, and 15-min intervals until metformin treatment. After being starved for 12 h, Huh-7 cells seeded in each plate were treated with the concentrations of 0- and 20-mM metformin dissolved in a complete DMEM. The impedance values were examined in 1-min intervals for 2 h, 5-min intervals for 2 h, and 15-min intervals for the remaining time. The cell index values based on impedance were normalized to the time point of metformin treatment and quantified by the RTCA software program.

### Cell Cycle Analysis

Huh-7 cells were planted into six-well plates at a concentration of 1.5 × 10^5^ cells per well with a 2-ml medium and maintained at 37°C and 5% CO_2_ for 24 h in a humid environment. After being starved in serum-free medium for 12 h, Huh-7 cells were treated with 0- and 20-mM concentrations of metformin for 48 h in a 37°C incubator and then harvested, washed by refrigerated phosphate-buffered saline, fixed in 70% refrigerated ethanol for 4 h at 4°C, and centrifuged at 1,500 rpm for 5 min. Subsequently, the fixed cells were washed by refrigerated phosphate-buffered saline, centrifuged at 1,500 rpm for 5 min and incubated with a mixture of 10-µl RNase, 25-µl propidium iodine (PI), and 500-µl dye buffer in the dark for 30 min at 37°C. Then, cell cycle distribution was analyzed by a fluorescence-activated cell sorting flow cytometer (BD Biosciences, NJ, United States) in G0/G1 phase, S phase, and G2/M phase.

### Total RNA Extraction

Huh-7 cells were planted in six-well plates at 1.5 × 10^5^ cells per well and treated with 0- and 20-mM metformin for 48 h after being starved. Total RNA was pooled from cells and extracted with TRIzol reagent following the detailed procedure provided by the manufacturer. The purity of RNA was assessed with a NanoPhotometer spectrophotometer (IMPLEN, CA, United States). The concentration of RNA was tested with Qubit 3.0 Fluorometer (Life Technologies, CA, United States). The integrity of RNA was confirmed with Agilent 2100 RNA Nano 6000 Assay Kit (Agilent Technologies, CA, United States). All samples in this study passed quality control with a minimum RNA integrity number of 8.0.

### Library Construction and RNA Sequencing

A transcriptome sequencing trial was carried out by ANOROAD (Beijing, China). Six cDNA libraries were constructed, *i.e.*, three for the 20-mM metformin group and three for the control group. After finishing libraries construction, qualified libraries were sequenced on an Illumina Hiseq platform.

### Data Analysis

Raw reads were obtained by converting original sequencing images to sequential reads. Clean reads were gained by completing data processing such as filtering low-quality reads, trimming adapter polluted reads, removing reads rich in ploy-N, and so on. The succeeding analyses were on the basis of high-quality clean reads after filtering. The genome version we referred to was Homo_sapiens.GRCh38.87.chr. Cufflinks v2.1.1 was used to analyze differentially expressed genes (DEGs) between the metformin and control groups. Transcript abundance representing the level of gene expression was examined by fragments per kilobase of exon model per million mapped fragments. Cuffdiff software was used to analyze DEGs according to the following three criteria: *1*) log2 fold change value > 1 or < −1; *2*) q-value false discovery rate < 0.05; and 3) fragments per kilobase of exon model per million mapped fragments value > 1.

### Functional Enrichment Analysis

Gene ontology (GO) functional enrichment analysis was carried out to interpret their biological functions to figure out the DEGs with metformin treatment. We used the GOseq R package to map all DEGs and to find significantly enriched GO terms by comparison with the genomic background and defined the GO terms with a corrected *p*-value of less than 0.05 to significantly enrich in DEGs. We used KOBAS software to perform Kyoto Encyclopedia of Genes and Genomes (KEGG) pathway enrichment analysis of DEGs compared with transcriptome background through a hypergeometric test. A hypergeometric *p* < 0.05 was considered to be significantly enriched.

### Quantitative Real-Time Polymerase Chain Reaction on Gene Expressions

The expression levels of total RNA from Huh-7 cells with metformin treatment for 48 h were detected by quantitative real-time (RT) PCR. Glyceraldehyde-3-phosphate dehydrogenase was used as a control gene. The primer sequences of the target and the control genes are shown in Table 1. Specific primers were selected from Gene Bank and synthesized by GENEWIZ (Suzhou Genomics Institute, Suzhou, China). Total RNA (1 μg) was extracted from treated Huh-7 cells using Trizol and reversely transcribed using the RT reagent kit. The real-time PCR reaction was carried out in a 10-μl buffer mixture composed of 5-μl AceQ qPCR SYBR Green Master Mix, 0.2 μM of each primer, 0.31-μl template cDNA, and 4.29-μl water free of nuclease. Thermal cycling was carried out following the instructions below: 95°C for 5 min, 40 cycles at 95°C for 10 s, and 60°C for 30 s. Quantitative RT-PCR assay was done in a 96-well optical plate (Roche, Light Cycler 96). The relative quantity of RNA transcript was worked out with the formula 2ΔCt (comparative cycling-threshold) that represents the difference between target gene expression in treated and control groups. The fold change of target gene cDNA relative to control gene was calculated: FC = 2^−ΔΔCt^.

**TABLE 1:** List of gene primers.

Gene	Forward primer	Reverse primer
FANS	5ʹ-ACA​GCG​GGG​AAT​GGG​TAC​T-3ʹ	5ʹ-GAC​TGG​TAC​AAC​GAG​CGG​AT-3ʹ
MCM6	5ʹ-TCG​GGC​CTT​GAA​AAC​ATT​CGT-3ʹ	5ʹ-TGT​GTC​TGG​TAG​GCA​GGT​CTT-3ʹ
MCM5	5ʹ-ATG​TCG​GGA​TTC​GAC​GAT​CCT-3ʹ	5ʹ-CCA​GGT​TGT​AAT​GCC​GCT​TG-3ʹ
PCNA	5ʹ-AGC​CTG​ACA​AAT​GCT​TGC​TGA​C-3ʹ	5ʹ-AGG​AAA​GTC​TAG​CTG​GTT​TCG​G-3ʹ
MARCKS	5ʹ-AGC​CCG​GTA​GAG​AAG​GAG​G-3ʹ	5ʹ-TTG​GGC​GAA​GAA​GTC​GAG​GA-3ʹ
FADS2	5ʹ-GAC​CAC​GGC​AAG​AAC​TCA​AAG-3ʹ	5ʹ-GAG​GGT​AGG​AAT​CCA​GCC​ATT-3ʹ
CXCL1	5ʹ-CAA​ACC​GAA​GTC​ATA​GCC​ACA​C-3ʹ	5ʹ-CTG​TTC​CTA​TAA​GGG​CAG​GGC-3ʹ
BMP4	5ʹ-ATG​ATT​CCT​GGT​AAC​CGA​ATG​C-3ʹ	5ʹ-CCC​CGT​CTC​AGG​TAT​CAA​ACT-3ʹ
SKP2	5ʹ-ATG​CCC​CAA​TCT​TGT​CCA​TCT-3ʹ	5ʹ-CAC​CGA​CTG​AGT​GAT​AGG​TGT-3ʹ
KNG1	5ʹ-TGC​TCC​AGG​CTG​CTA​CTA​AGT-3ʹ	5ʹ-GGC​TTC​AGT​TAT​GCG​GTA​CAA-3ʹ
DUSP1	5ʹ-TCG​AGA​GGG​CTG​GTC​CTT​AT-3ʹ	5ʹ-GGG​GCG​AGC​AAA​AAG​AAA​CC-3ʹ

### Statistical Analysis

All data of each group were analyzed by mean ± standard deviation for at least three independent trials using SPSS software version 16.0. Unpaired Student's t-test or Mann–Whitney U test was applied to compare the difference between two groups. One-way analysis of variance and Tukey's *post-hoc* test were applied to compare the multiple groups' differences. The difference has a statistical significance when the *p*-value is less than 0.05.

## Results

### Metformin Inhibits the Proliferation of Human Liver Cancer Huh-7 Cells

**FIGURE 1:**
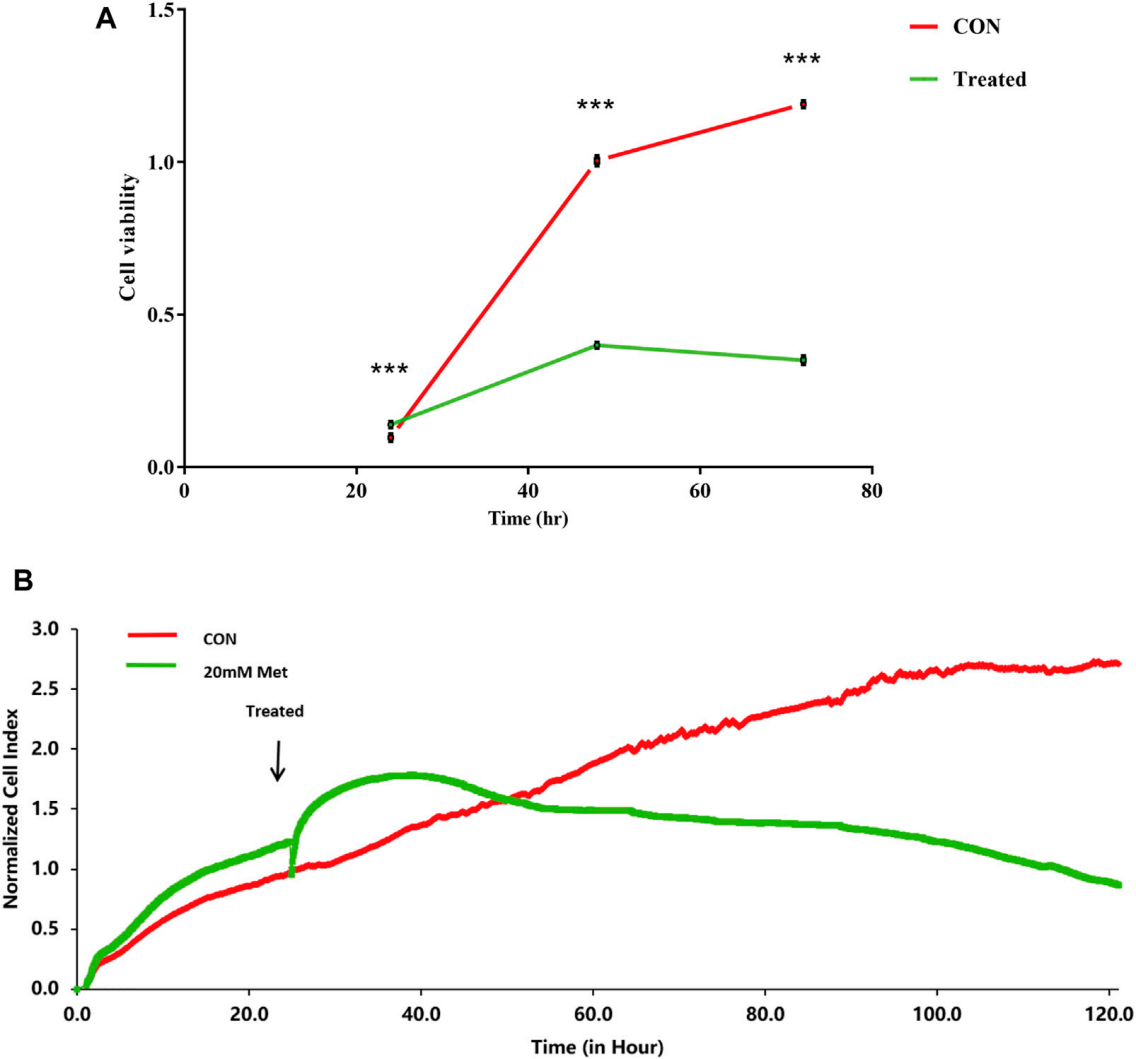
Effect of metformin (Met) on cell growth in Huh-7 cells. **(A)** Cell proliferation was tested through MTT assay. Huh-7 Cells were handled with 20-mM Met for 24, 48, and 72 h, and cell proliferation was tested by MTT reduction assay. Data are presented by mean ± standard deviation of five independent experiments performed in triplicates. ***p < 0.001, **p < 0.01, *p < 0.05 *versus* CON without metformin. **(B)** Real-time monitoring of proliferation of Huh-7 cells on RTCES platform. Cells suspensions were added into E-Plates and laid on RTCES reader to real-timely monitor every 30 min during assay. Arrow denotes point-in-time of adding metformin.

Previous studies have shown that metformin has an inhibiting effect in various cancer cells (Hsieh et al., 2014; Shi et al., 2017; Yu et al., 2017). To inspect the effect of metformin on the proliferation of Huh-7 cells, an MTT assay was utilized to examine cell viability. The results revealed that metformin inhibited the proliferation of Huh-7 cells in a time-dependent manner treated with 0- and 20-mM metformin for 24, 48, and 72 h (Figure 1A).

### Time-Dependent Cell Response Analysis of Metformin by Real-Time Cell Analyzer

RTCA is applied to monitor the effect of small molecule compounds and approved drugs on a cell culture based on impedance readout of time-dependent cell response profiles (TCRPs). The utility of this approach is to test how a bioactive compound affects cell proliferation, migration, infiltration, and intercellular interaction. The research paper described that compounds possessed similar activity if they had displayed similar TCRPs based on impedance, and the mechanism of small molecule compounds might be predicted through the TCRP method (Abassi et al., 2009). Our study showed that a bell-shaped TCRP was tested after metformin treatment and the cell index first increased and then decreased compared with the control group, which reflected a final cytotoxic reaction (Figure 1B). In Abassi et al.'s study, bell-shaped TCRP suggested that metformin might interfere with DNA unwinding, replication, synthesis, transcription, and translation, which is in accordance with our transcriptome outcome.

### Metformin Inhibits Cell Cycle Progression in Human Liver Cancer Cells

**FIGURE 2:**
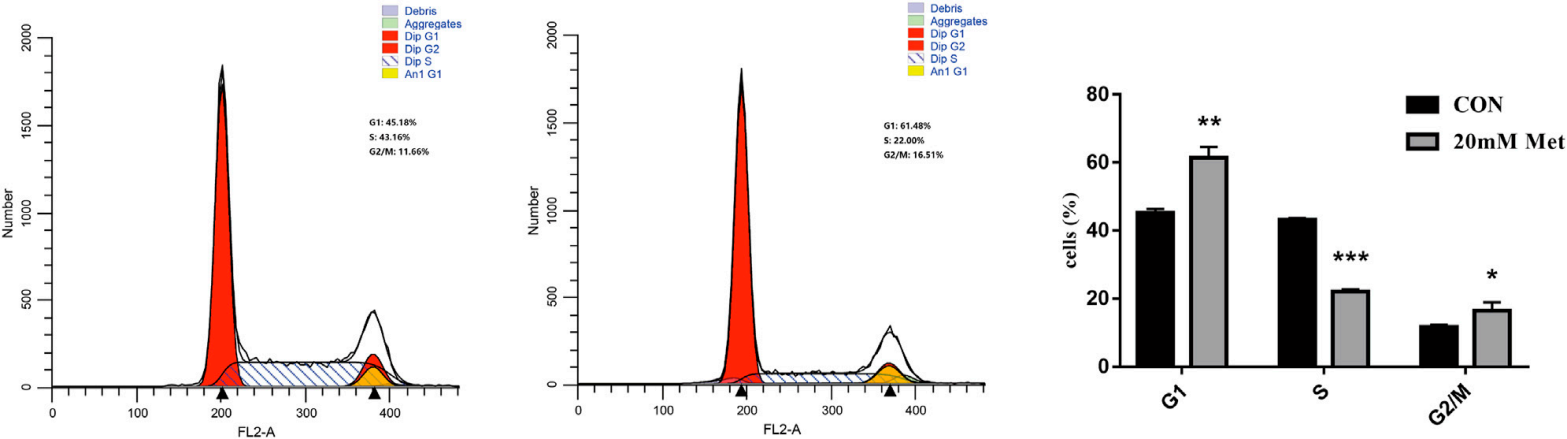
Effect of metformin on cell cycle in Huh-7 cells. Cells were treated with 20-mM Met for 48 h and analyzed for propidium iodide-stained DNA content using flow cytometry. Column diagram shows result of statistical analysis. ***p < 0.001, **p < 0.01, *p < 0.05 *versus* CON without metformin.

The cell cycle is composed of a range of tightly controlled events that take place in a cell and drive the replication of DNA and cell division. It was reported that the abnormal control of the cell cycle might be closely associated with the occurrence and development of tumors (Kastan and Bartek, 2004; Ingham and Schwartz, 2017). Therefore, to investigate the effect of pharmacological intervention on the cell cycle is developing into a potential pathway for tumor treatment. To further explore the mechanisms of the cell growth inhibition induced by metformin, we carried out a flow cytometry analysis to inspect alterations of the cell cycle after metformin treatment on Huh-7 cells for 48 h. As shown in Figure 2 **,** metformin could have a significant effect on the cell cycle distribution. Compared with the CON group, 20-mM metformin increased the G1 proportion of Huh-7 cells from 45.18 to 61. 48% and decreased S-phase proportion of cell cycle from 43.16 to 22.00%. These results suggested that metformin inhibited Huh-7 cells growth through arresting cells at the G1 phase.

### RNA Sequencing Transcriptome Analysis of Metformin Treatment

**FIGURE 3:**
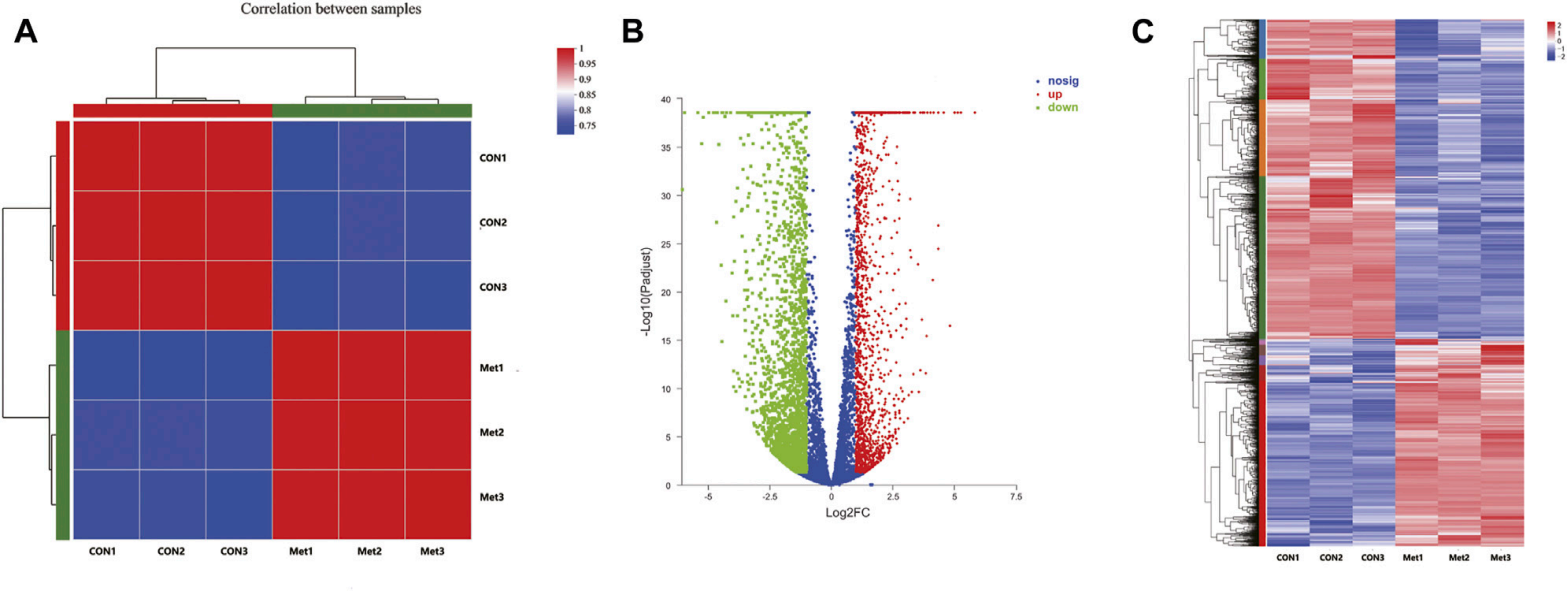
Differentially expressed genes in Huh-7 cells treated with 20-mM metformin for 48 h. **(A)** Correlation coefficient plot. Right and bottom of plot represent sample names, and left and top of plot represent sample clustering. Higher value of correlation coefficient, greater correlation between two samples. **(B)** Volcano map. *x*-Axis indicates log fold change of differential expression genes between treatment and control groups, and *y*-axis indicates −log 10 of adjusted *p*-value. Each point represents a gene. Red points present significantly upregulated genes, green points present significantly downregulated genes, and blue dots were genes with not significantly different expression, which met screening condition of FC > 1, adjusted *p*-value (adj *P*) < 0.05. **(C)** Differential genes cluster heat map. It was a hierarchical clustering analysis of up- and downregulated genes. Red and purple, respectively, indicate up- and downregulated genes.

To further investigate the antitumor biological mechanisms involved with Huh-7 cells treated with metformin, RNA sequencing was performed to detect the effect of metformin on the transcriptome with or without treatment of 20-mM metformin for 48 h in triplicate. A total of 37.24-Gb clean bases were obtained, of which 18.53 Gb from the control group (0-mM metformin, three replicates) and 18.71 Gb from the treatment group (20-mM metformin, three replicates). The clean data of all samples reached more than 6.52 Gb, and the percentage of Q30 base was more than 96%. Then, these clean reads of the two groups were mapped to reference genomes version GRCh38 from Homo_sapiens for quantitative measurement of gene expression, and the mapped rate ranged from 98.15 to 98.56%, indicating high levels of gene expression in all samples. A total of 4,518 DEGs were obtained based on screening conditions of adjusted p-value < 0.05 and |log2FoldChange| > 1 with or without metformin treatment in Huh-7 cells**.** Among those genes, 1,812 were upregulated, and 2,706 were downregulated (Figures 3A–C). Some significant genes are shown in Tables 2, 3. Those genes were related to such signaling pathways as cell cycle, cell communication, cell adhesion, blood vessel morphogenesis, extracellular matrix organization, DNA replication, positive regulation of phosphorylation, and so on.

**TABLE 2:** Twenty significantly upregulated genes linked to cancer in Huh-7 cells treated with metformin.

Gene_ID	Gene name	Gene description	log2FoldChange	Adjusted P-value
ENSG00000136997	MYC	MYC proto-oncogene, bHLH transcription factor	2.98	4.56E−209
ENSG00000223802	CERS1	ceramide synthase 1	2.68	6.05E−22
ENSG00000157613	CREB3L1	cAMP responsive element binding protein 3 like 1	2.42	1.17E−77
ENSG00000113070	HBEGF	heparin-binding EGF like growth factor	2.42	1.11E−30
ENSG00000164136	IL15	interleukin 15	2.40	1.82E−33
ENSG00000120129	DUSP1	dual specificity phosphatase 1	2.39	1.14E−159
ENSG00000185950	IRS2	insulin receptor substrate 2	2.22	1.03E−100
ENSG00000060566	CREB3L3	cAMP responsive element binding protein 3 like 3	2.06	1.40E−60
ENSG00000112715	VEGFA	vascular endothelial growth factor A	1.96	2.70E−130
ENSG00000177606	JUN	Jun proto-oncogene, AP-1 transcription factor subunit	1.82	3.82E−67
ENSG00000066027	PPP2R5A	protein phosphatase 2 regulatory subunit B’alpha	1.76	8.16E−135
ENSG00000160741	CRTC2	CREB regulated transcription coactivator 2	1.75	3.12E−76
ENSG00000096717	SIRT1	sirtuin 1	1.58	6.64E−50
ENSG00000254413	CHKB-CPT1B	CHKB-CPT1B readthrough (NMD candidate)	1.46	4.14E−65
ENSG00000105499	PLA2G4C	phospholipase A2 group IVC	1.45	4.08E−11
ENSG00000146592	CREB5	cAMP responsive element binding protein 5	1.36	4.39E−25
ENSG00000110090	CPT1A	carnitine palmitoyltransferase 1A	1.25	4.77E−51
ENSG00000167658	EEF2	eukaryotic translation elongation factor 2	1.19	1.36E−74
ENSG00000205560	CPT1B	carnitine palmitoyltransferase 1B	“”1.18	4.32E−18
ENSG00000132170	PPARG	peroxisome proliferator activated receptor gamma	1.17	1.92E−27

**TABLE 3:** Twenty significantly downregulated genes linked to cancer in Huh-7 cells treated with metformin.

Gene_ID	Gene name	Gene description	log2FoldChange	Adjusted P-value
ENSG00000112486	CCR6	C-C motif chemokine receptor 6	−9.61	1.18E−14
ENSG00000143847	PPFIA4	PTPRF interacting protein alpha 4	−6.17	2.66E−25
ENSG00000163739	CXCL1	C-X-C motif chemokine ligand 1	−6.27	8.73E−90
ENSG00000261787	TCF24	transcription factor 24	−5.93	1.42E−04
ENSG00000134824	FADS2	fatty acid desaturase 2	−3.22	3.26E−244
ENSG00000113889	KNG1	kininogen 1	−3.12	6.13E−77
ENSG00000134853	PDGFRA	platelet derived growth factor receptor alpha	−3.08	9.92E−05
ENSG00000107984	DKK1	dickkopf WNT signaling pathway inhibitor 1	−2.37	1.94E−01
ENSG00000132646	PCNA	proliferating cell nuclear antigen	−2.31	1.18E−167
ENSG00000125378	BMP4	bone morphogenetic protein 4	−2.12	1.01E−66
ENSG00000145604	SKP2	S-phase kinase associated protein 2	−2.03	5.00E−143
ENSG00000277443	MARCKS	myristoylated alanine rich protein kinase C substrate	−2.00	1.38E−61
ENSG00000100297	MCM5	minichromosome maintenance complex component 5	−1.95	1.26E−94
ENSG00000076003	MCM6	minichromosome maintenance complex component 6	−1.90	1.26E−67
ENSG00000169710	FASN	fatty acid synthase	−1.64	3.38E−61
ENSG00000104812	GYS1	glycogen synthase 1	−1.40	—
ENSG00000137713	PPP2R1B	protein phosphatase 2 scaffold subunit Abeta	−1.38	7.40E−31
ENSG00000167244	IGF2	insulin like growth factor 2	−1.23	4.13E−73
ENSG00000111665	CDCA3	cell division cycle associated 3	−1.16	4.85E−31
ENSG00000182158	CREB3L2	cAMP responsive element binding protein 3 like 2	−1.11	1.51E−34

### Gene Ontology Analysis of Differentially Expressed Genes

**FIGURE 4:**
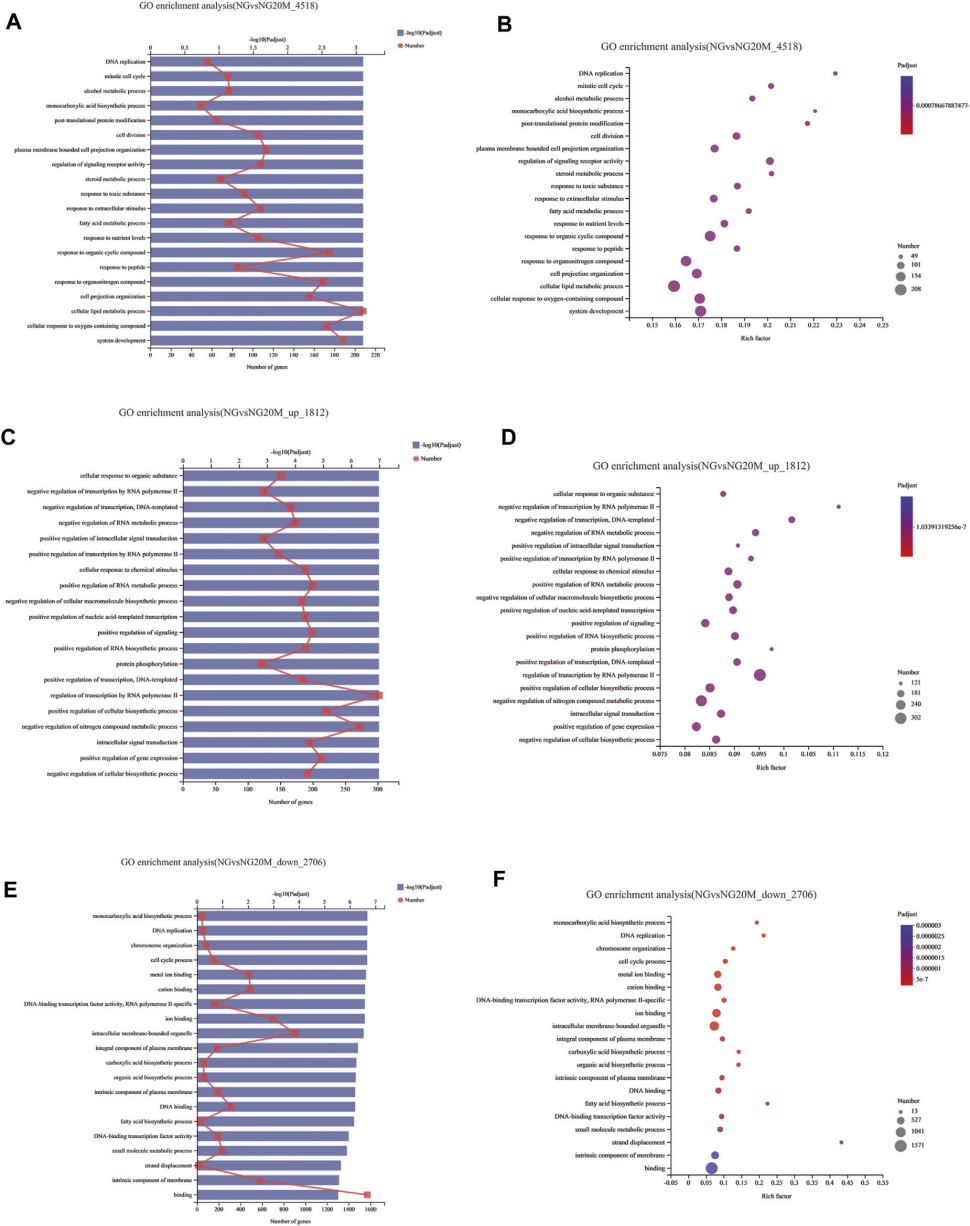
GO enrichment analysis of DEGs in metformin-treated Huh-7. **(A)** Gene ontology (GO) functional classification on all DEGs between control group and metformin treatment group. **(B)** Bubble chart of all DEG GO analyses. **(C)** GO functional classification on upregulated DEGs. **(D)** Bubble chart of upregulated DEG GO analyses. **(E)** GO functional classification on downregulated DEGs. **(F)** Bubble chart of downregulated DEG GO analyses. In column charts, upper *x*-axis indicates quantity of genes corresponding to different points on fold line; lower *x*-axis indicates negative logarithm of adjusted p-value of GO terms representing significance enrichment level corresponding to height of column; *y*-axis indicates GO terms. In bubble charts, *y*-axis represents GO terms; *x*-axis indicates Rich factor, which is ratio of sample quantity enriched in GO term to background quantity of annotated genes. Bigger Rich factor, higher enrichment degree. Dot dimension represents quantity of genes of GO terms, and dot color corresponds to different *p*-value ranges.

To illustrate the functional changes of gene expression associated with metformin treatment in Huh-7 cells, a GO enrichment analysis of 4,518 DEGs from three libraries was carried out. We derived 575 enriched GO terms (q < 0.05), 430 categories in biological processes, 58 categories in cellular components, and 87 categories in molecular functions. The significant enrichment biological processes included cell cycle, cellular response to chemical stimulus, regulation of signaling, biological adhesion, positive regulation of phosphorylation, blood vessel morphogenesis, cell population proliferation, regulation of cell adhesion, and so on (Figures 4A,B and Table 4). Meanwhile, we performed GO enrichment analysis of upregulated and downregulated DEGs. The downregulated genes were significantly enriched in 212 GO terms (q < 0.05), which are 146 categories in biological process, 29 categories in a cellular component, and 37 categories in molecular function. The top of the ranking categories in the biological process included DNA replication, fatty acid (FA) biosynthetic process, steroid metabolic process, mitotic cell cycle, and so on (Figures 4C,D and Table 5). The upregulated genes were significantly enriched in 618 GO terms (q < 0.05), among which 469 in biological process, 44 in a cellular component, and 105 in molecular function. The top of the ranking list in biological process with upregulated genes included regulation of positive regulation of intracellular signal transduction, cell surface receptor signaling pathway, response to cytokine, and so on (Figures 4E,F and Table 6). The enrichment degree of membrane transport and molecular regulation was the highest, and the enrichment rate of protein binding was the highest.

**TABLE 4:** List of 20 biological process entries for all differentially expressed genes.

GO ID	Description	Number	Adjusted P-value
GO:0007049	cell cycle	172	0.00079
GO:0022402	cell cycle process	230	0.00079
GO:0070887	cellular response to chemical stimulus	337	0.00079
GO:0042325	regulation of phosphorylation	328	0.00079
GO:0035556	intracellular signal transduction	317	0.00079
GO:0007154	cell communication	167	0.00079
GO:0023051	regulation of signaling	686	0.00080
GO:0009967	positive regulation of signal transduction	311	0.00080
GO:0022610	biological adhesion	176	0.00082
GO:0030198	extracellular matrix organization	77	0.00083
GO:0042326	negative regulation of phosphorylation	108	0.00085
GO:0034097	response to cytokine	119	0.00085
GO:0007155	cell adhesion	173	0.00098
GO:0043408	regulation of MAPK cascade	159	0.00148
GO:0023052	signaling	127	0.00236
GO:0042327	positive regulation of phosphorylation	210	0.00275
GO:0008283	cell population proliferation	151	0.00338
GO:0030155	regulation of cell adhesion	151	0.00407
GO:0007267	cell-cell signaling	113	0.00413
GO:0048514	blood vessel morphogenesis	26	0.00693

**TABLE 5:** List of 20 biological process entries for upregulating differentially expressed genes.

GO ID	Description	Number	Adjusted P-value
GO:1902533	positive regulation of intracellular signal transduction	124	1.03E−07
GO:1902531	regulation of intracellular signal transduction	218	1.03E−07
GO:0009967	positive regulation of signal transduction	188	1.03E−07
GO:0048584	positive regulation of response to stimulus	244	1.03E−07
GO:0009966	regulation of signal transduction	349	1.03E−07
GO:0051716	cellular response to stimulus	314	1.03E−07
GO:0023051	regulation of signaling	376	1.03E−07
GO:0010646	regulation of cell communication	371	1.03E−07
GO:0032879	regulation of localization	269	1.04E−07
GO:0001932	regulation of protein phosphorylation	163	1.26E−07
GO:0043408	regulation of MAPK cascade	91	8.58E−06
GO:0007166	cell surface receptor signaling pathway	220	1.08E−05
GO:0043405	regulation of MAP kinase activity	48	3.11E−05
GO:0010942	positive regulation of cell death	85	3.67E−05
GO:0043065	positive regulation of apoptotic process	80	5.13E−05
GO:0042981	regulation of apoptotic process	164	5.36E−05
GO:0080135	regulation of cellular response to stress	86	5.36E−05
GO:0001817	regulation of cytokine production	78	7.97E−05
GO:0034097	response to cytokine	66	0.00012
GO:0030155	regulation of cell adhesion	83	0.00027

**TABLE 6:** List of 20 biological process entries for downregulating differentially expressed genes.

GO ID	Description	Number	Adjusted P-value
GO:0072330	monocarboxylic acid biosynthetic process	43	2.08E−07
GO:0006260	DNA replication	52	2.08E−07
GO:0051276	chromosome organization	84	2.12E−07
GO:0022402	cell cycle process	152	2.12E−07
GO:0006633	fatty acid biosynthetic process	28	6.93E−07
GO:0098742	cell-cell adhesion *via* plasma-membrane adhesion molecules	46	5.50E−06
GO:1903047	mitotic cell cycle process	98	1.57E−05
GO:0007049	cell cycle	94	0.00076
GO:0008202	steroid metabolic process	43	0.00087
GO:0030195	negative regulation of blood coagulation	14	0.00131
GO:0000278	mitotic cell cycle	46	0.00139
GO:0007155	cell adhesion	105	0.00329
GO:0098609	cell-cell adhesion	63	0.00341
GO:0000082	G1/S transition of mitotic cell cycle	21	0.00341
GO:0044843	cell cycle G1/S phase transition	21	0.00376
GO:0010469	regulation of signaling receptor activity	55	0.01937
GO:0007267	cell-cell signaling	67	0.03282
GO:0023052	signaling	74	0.03832
GO:0055091	phospholipid homeostasis	5	0.04751
GO:0030193	regulation of blood coagulation	16	0.04778

KEGG pathway analysis of differentially expressed genes.

**FIGURE 5:**
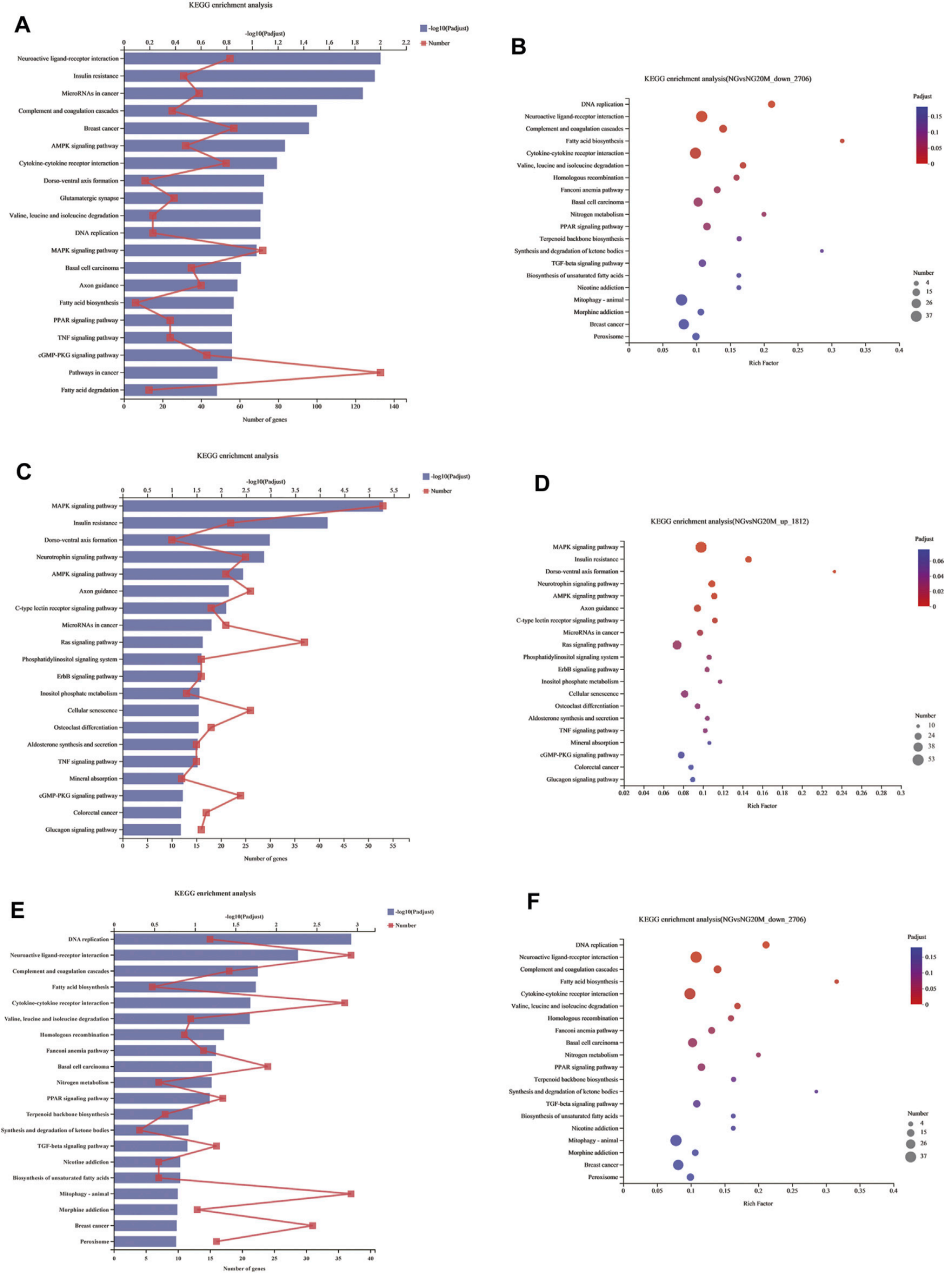
KEGG enrichment analysis of DEGs. **(A)** KEGG enrichment analysis of all DEGs. **(B)** Bubble chart of all DEGs about KEGG enrichment analysis. **(C)** KEGG enrichment analysis of upregulated DEGs. **(D)** Bubble chart of upregulated DEGs about KEGG enrichment analysis. **(E)** KEGG enrichment analysis of downregulated DEGs. **(F)** Bubble chart of downregulated DEGs about KEGG enrichment analysis. In column charts, lower *x*-axis represents number of genes compared with this pathway, corresponding to different dots on fold line; upper *x*-axis indicates significance enrichment level, corresponding to height of column. Smaller FDR is, bigger −log10 (q value) is, and more significantly enriched KEGG pathway is. In bubble charts, *y*-axis represents KEGG pathways and their names; *x*-axis indicates Rich factor, which is ratio of sample quantity enriched in this pathway to background quantity. Bigger Rich factor, higher enrichment degree. Dot dimension represents gene quantity, and dot color corresponds to different *p*-value ranges.

Genes in an organism mutually coordinate to perform biological functions, and pathway analysis is conducive to further understanding them. To identify molecular mechanisms of metformin acted on Huh-7 cells, the 4,518 DEGs were subjected to KEGG enrichment analysis. As a consequence, 54 KEGG pathways (*p* < 0.05) (Figures 5A,B and Table 7) were significantly differentially enriched, which included microRNAs in cancer, AMPK signaling pathway, cytokine–cytokine receptor interaction, complement and coagulation cascades, TNF signaling pathway, pathways in cancer, FA biosynthesis, basal cell carcinoma, cGMP-PKG signaling pathway, PPAR signaling pathway, and so on. The 1,812 upregulated genes were significantly enriched in 63 KEGG pathways (*p* < 0.05) (Figures 5C,D and Table 8), which included MAPK signaling pathway, AMPK signaling pathway, Ras signaling pathway, phosphatidylinositol signaling system, ErbB signaling pathway, inositol phosphate metabolism, TNF signaling pathway, Hippo signaling pathway—multiple species, FoxO signaling pathway, aldosterone synthesis and secretion, Jak-STAT signaling pathway, PI3K-Akt signaling pathway, and so on. The 2,706 downregulated genes were significantly enriched in 41 KEGG pathways (*p* < 0.05) (Figures 5E,F and Table 9), which included DNA replication, cell cycle, FA biosynthesis, valine, leucine and isoleucine degradation, extracellular matrix (ECM)–receptor interaction, transforming growth factor (TGF)-β signaling pathway, Fanconi anemia pathway, basal cell carcinoma, synthesis and degradation of ketone bodies, and so on. In these pathways, we paid most attention to these pathways, such as DNA replication, cell cycle, and ECM–receptor interaction.

**TABLE 7:** List of 20 KEGG analyses of all differentially expressed genes.

Term	ID	Input number	Background number	*p*-value
Neuroactive ligand-receptor interaction	map04080	55	343	8.47E−05
Insulin resistance	map04931	31	151	3.13E−05
MicroRNAs in cancer	map05206	39	217	7.74E−05
Complement and coagulation cascades	map04610	25	129	0.00044
Breast cancer	map05224	57	381	0.00041
AMPK signaling pathway	map04152	32	189	0.00094
Cytokine-cytokine receptor interaction	map04060	53	365	0.00127
Dorso-ventral axis formation	map04320	11	43	0.00183
Glutamatergic synapse	map04724	26	154	0.00280
Valine, leucine and isoleucine degradation	map00280	15	71	0.00243
DNA replication	map03030	15	71	0.00243
MAPK signaling pathway	map04010	72	542	0.00235
Basal cell carcinoma	map05217	35	234	0.00484
Axon guidance	map04360	40	276	0.00479
Fatty acid biosynthesis	map00061	6	19	0.00670
PPAR signaling pathway	map03320	24	147	0.00610
TNF signaling pathway	map04668	24	147	0.00610
cGMP-PKG signaling pathway	map04022	43	309	0.00733
Pathways in cancer	map05200	133	1,166	0.01530
Fatty acid degradation	map00071	13	70	0.01384

**TABLE 8:** List of 20 KEGG analyses of upregulated differentially expressed genes.

Term	ID	Input number	Background number	*p*-value
MAPK signaling pathway	map04010	53	542	1.60E−08
Insulin resistance	map04931	22	151	4.26E−07
Dorso-ventral axis formation	map04320	10	43	9.52E−06
Neurotrophin signaling pathway	map04722	25	230	1.66E−05
AMPK signaling pathway	map04152	21	189	5.54E−05
Axon guidance	map04360	26	276	0.00013
C-type lectin receptor signaling pathway	map04625	18	161	0.00017
MicroRNAs in cancer	map05206	21	217	0.00038
Ras signaling pathway	map04014	37	503	0.00087
Phosphatidylinositol signaling system	map04070	16	151	0.00069
ErbB signaling pathway	map04012	16	154	0.00086
Inositol phosphate metabolism	map00562	13	111	0.00084
Cellular senescence	map04218	26	320	0.00123
Osteoclast differentiation	map04380	18	191	0.00132
Aldosterone synthesis and secretion	map04925	15	144	0.00119
TNF signaling pathway	map04668	15	147	0.00147
Mineral absorption	map04978	12	113	0.00303
cGMP-PKG signaling pathway	map04022	24	309	0.00332
Colorectal cancer	map05210	17	194	0.00378
Glucagon signaling pathway	map04922	16	179	0.00402

**TABLE 9:** List of 20 KEGG analyses of downregulated differentially expressed genes.

Term	ID	Input number	Background number	*p*-value
DNA replication	map03030	15	71	3.55E−06
Neuroactive ligand-receptor interaction	map04080	37	343	3.22E−05
Complement and coagulation cascades	map04610	18	129	0.00015
Fatty acid biosynthesis	map00061	6	19	0.00032
Cytokine-cytokine receptor interaction	map04060	36	365	0.00025
Valine, leucine and isoleucine degradation	map00280	12	71	0.00031
Homologous recombination	map03440	11	69	0.00092
Fanconi anemia pathway	map03460	14	107	0.00149
Basal cell carcinoma	map05217	24	234	0.00148
Nitrogen metabolism	map00910	7	35	0.00205
PPAR signaling pathway	map03320	17	147	0.00197
Terpenoid backbone biosynthesis	map00900	8	49	0.00384
Synthesis and degradation of ketone bodies	map00072	4	14	0.00504
TGF-beta signaling pathway	map04350	16	147	0.00480
Nicotine addiction	map05033	7	43	0.00680
Biosynthesis of unsaturated fatty acids	map01040	7	43	0.00680
Mitophagy - animal	map04137	37	474	0.01166
Morphine addiction	map05032	13	122	0.01228
Breast cancer	map05224	31	381	0.01153
Peroxisome	map04146	16	161	0.01123

### Quantitative Real-Time Polymerase Chain Reaction Validation

**FIGURE 6:**
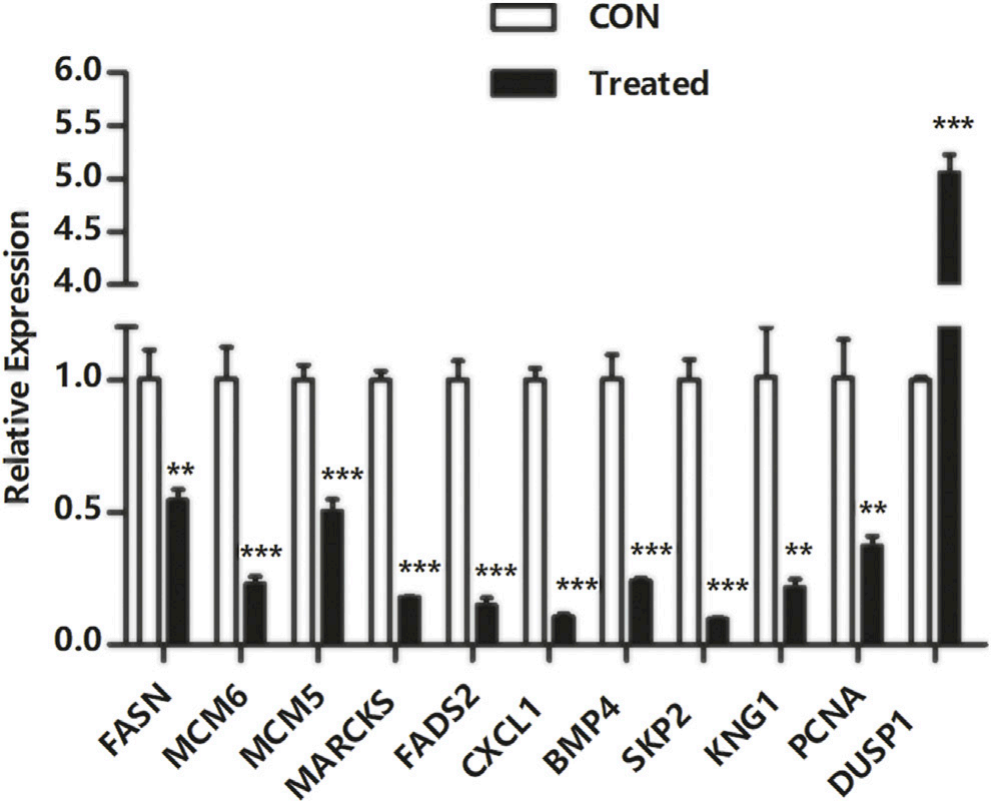
Quantitative real-time polymerase chain reaction validation of DEGs. Total RNA was isolated from Huh-7 cells treated with 20-mM metformin and carried out a quantitative real-time polymerase chain reaction trial in triplicate. Glyceraldehyde-3-phosphate dehydrogenase was a reference gene. Use a comparative threshold cycle (Ct) method to analyze data. *p < 0.05,**p < 0.01, ***p < 0.001 *versus* CON without metformin.

To verify the transcriptome sequencing outcome and to further investigate gene expression pattern associated with cancer, 11 representative genes were selected for quantitative RT-PCR from KEGG data, including microRNAs in cancer pathway [myristoylated alanine-rich C-kinase substrate (MARCKS)], DNA replication pathway [mini-chromosome maintenance complex component 6 (MCM6), MCM5, and proliferating cell nuclear antigen (PCNA)], pathway in cancer [bone morphogenetic protein 4 (BMP4) and S-phase kinase-associated protein 2 (SKP2)], complement and coagulation cascades [kininogen 1 (KNG1)], FA biosynthesis [fatty acid synthase (FASN)], MAPK pathway [dual-specificity phosphatase-1 (DUSP1)], PPAR signaling pathway [fatty acid desaturase 2 (FADS2)], and TNF signaling pathway [C-X-C motif chemokine ligand 1 (CXCL1)]. The RT-PCR results showed that Huh-7 cells treated with metformin compared with the control group had much higher expression of DUSP1 (*p* < 0.05) but lower expressions of FASN, MCM6, MCM5, MARCKS, FADS2, CXCL1, BMP4, SKP2, KNG1, and PCNA (*p* < 0.05), which was consistent with the transcriptome sequencing outcome (Figure 6).

## Discussion

Human liver cancer cell lines are commonly used for the detection of anticancer drugs and the research on physiological characteristics of liver cells because they are derived from the tissues of hepatocarcinoma patients, have a set of complete genes, and are able to be relatively stably passaged. The widely used liver cell lines contain Huh-7, HepG-2, SMMC-7721, Bel-7402, and so on. Huh-7 cell line possessing the properties of being positive for AFP (Nakabayashi et al., 1985) and negative for HBV and susceptible to HCV (Sainz et al., 2009) compared with other liver cancer lines can be applied to study the regulation mechanism of gene expression, metabolism, xenotransplantation animal models, and so on.

Transcriptome analysis has been used to identify a potential mechanism of drug action on cancer from the perspective of functional genes and pathway networks (Abaan et al., 2013). An investigative transcriptome analysis was carried out to elucidate an underlying action mechanism of metformin in liver cancer cells. We discovered probable gene targets of metformin through researching differential gene expression and described underlying signaling pathways induced by metformin. Although further investigation is needed to verify these findings, this study may still supply a new perspective for functional validation of succeeding trials. The occurrence and development of cancer are complicated processes involving gene changes. The analysis of participation of a variety of signal molecules in the biological process helps to clarify metformin's antitumor mechanism. The transcriptome analysis revealed that loads of genes were significantly altered in Huh-7 cells with treatment of metformin, implying that these significantly changed genes might play a significant role in the antitumor effect of metformin *in vitro*. These genes involved 10 downregulated ones and 1 upregulated one in Huh-7 cells treated with metformin.

DUSP1 is a kind of phosphatase and can negatively regulate the activation of JNKs, ERKs, and p38 MAPK, which plays an important role in the regulation of the human cell growth cycle and tumor genesis and development through signaling pathways such as the MAPK pathway (Ueda et al., 2003). In this research, the expression of DUSP1 was upregulated in Huh-7 cells after metformin treatment. Previous studies showed that the DUSP1 gene, as a transcriptional target of the p53 tumor suppressor, is highly expressed in prostate cancer (Hou et al., 2012) and played a vital role to inhibit tumor progression in these tumors such as prostate cancer, bladder cancer, and colon cancer (Slattery et al., 2012).

In addition, some genes were significantly downregulated in Huh-7 cells treated with metformin, including FASN, MCM5, MCM6, PCNA, MARCKS, FADS2, CXCL1, BMP4, SKP2, and KNG1.

FASN is a critical enzyme of synthetizing long-chain FAs from malonyl-CoA and acetyl-coenzyme A (CoA) and has been identified at high levels in several human cancers such as prostate cancer (Souchek et al., 2017), pancreatic cancer (Alo et al., 2007), breast cancer (Gonzalez-Guerrico et al., 2016), and colorectal cancer (Zaytseva et al., 2015). FASN overexpression in cancers had frequently been related to biological aggressiveness and a poor prognosis (Gansler et al., 1997). Some studies revealed that FASN overexpression was closely associated with the PI3K-AKT pathway and SREBP 1c transcriptional regulation. Therefore, FASN was considered one of the more than 600 most promising therapeutic drug targets in human cancers (Behan et al., 2019). Proteins of the mini-chromosome maintenance family, including MCM2–7, are collectively referred to as MCM, which mainly affect the stability of microchromosome mitosis. MCM5 and MCM6, as two members of the MCM family, are associated with the initiation and extension of DNA replication and cell proliferation (Semple and Duncker, 2004). MCM5 overexpression has been discovered in some human cancers containing cervical cancer, bladder cancer, and so on (Alison et al., 2002). Therefore, MCM proteins could always be identified as a biomarker for tumor cells that might improve diagnostic accuracy. PCNA is a kind of nuclear protein that ubiquitously exists in normal proliferating cells and tumor cells. PCNA synthesized during all stages of the cell cycle has a crucial effect on replicating and repairing DNA through supplying DNA polymerases, and loads of protein partners participated in some significant biological processes such as DNA repair and cell cycle (Naryzhny, 2008). PCNA is an indispensable constituent of the ultimate mutual pathway shared by all mitogenic signals and is always in a central position for numerous other cell-signaling pathways. PCNA also has a high level of expression in numerous tumor cells. Therefore, PCNA is identified as a potentially valuable biomarker for cancer diagnosis and prognostic therapeutics (Naryzhny and Lee, 2007). MARCKS, a non-helical, nonspherical, and nonstructural acidic protein, is mainly a kind of PKC substrates taking part in various biological processes involving cytoskeletal control, cell cycle regulation, chemotactic effect, inflammatory reaction, and so on (Lee et al., 2015). In different physiological environments, MARCKS interacts with signals of downstream pathways. Abnormal MARCKS signals may participate in malignant deformation, uncontrolled proliferation, migration, and invasion of malignant tumor cells. Recently, some studies showed that as a proto-oncogene, MARCKS takes part in the tumorigenesis and development of several cancers such as lung cancer (Rohrbach et al., 2015), breast cancer (Chen et al., 2015), renal cell carcinoma (Chen et al., 2017), pancreatic cancer (Brandi et al., 2016), and HCC (Naboulsi et al., 2016) and plays an important role in participation in signaling pathways of many cancers. In addition, MARCKS also has an effect on the sensitivity of cancer cells to chemotherapy drugs and targeted radiation therapy. For these reasons, MARCKS might be an underlying drug target (Chen et al., 2014). FADS2 is one of the FA desaturase gene families.

FADS2 (Ralston et al., 2015), encoding a membrane-binding protein composed of 444 amino acids that cross the cytomembrane four times (Marquardt et al., 2000), has a vital effect on the biosynthesis process of FA (Nakamura and Nara, 2004). The main function of FADS2 is to regulate the synthesis of polyunsaturated fatty acids by introducing a double bond in the hydrocarbon chain of FAs. FADS2 is the first rate-limiting enzyme in polyunsaturated fatty acid synthesis (Lee and Park, 2014). Recent studies showed that FADS2 has a high expression level in breast cancer tissues and esophageal squamous cell carcinoma, which plays a vital role in the occurrence and development of these cancers (Lagarde and Nicolaou, 2015). FADS2 has become a promising drug target in the treatment of cancers. CXCL1, a small molecular cytokine, belongs to the CXC chemokine family, also known as growth regulating oncogene a (GRO a). It produces normal physiological and immune responses by recruiting specific cell groups to infect the specific site of the tumor (Yapa et al., 2017). CXCL1 not only plays a significant role in inflammatory response but also participates in a variety of biological processes, such as angiogenesis tumor genesis (He et al., 2012), atherosclerosis (Boisvert et al., 1998), wound healing, and so on (Ridiandries et al., 2017). Some studies showed that several liver diseases could induce the high expression of CXCL1 in hepatocytes and hepatic astrocytes and eventually lead to tumor genesis, development, invasion, metastasis, and poor prognosis (Miyake et al., 2013). It has also been reported that in the microenvironment of liver cancer, hepatic astrocytes could secrete CXCL1, which helps to induce epithelial–mesenchymal transformation of hepatocarcinoma cells, resistance to regulation, migration, and invasion, and degradation of extracellular matrix by activating the PI3K/AKT pathway (Sobolik et al., 2014). Because of playing an important role in tumor development and potential clinical significance, as a tumor marker, CXCLl might be considered as a therapeutic target of cancer. BMP4, belonging to the BMP subfamily, is a kind of multifunctional signaling molecule that plays a crucial role in regulating important biological functions such as cell growth and development, tissue differentiation, organ formation, apoptosis, and so on (Watanabe et al., 2001). As an upstream signaling molecule, BMP4 carries on its biological functions mainly by activating the intracellular downstream signaling pathway. Some studies showed that in addition to Smad signaling pathway in response to BMPs, BMP4 could also activate downstream p38, JNK, ERK1/2, and other kinases through the interaction between the receptor and MAPKKK, and then p38, JNK, ERK1/2, and other signaling pathways are activated to regulate the transcription of downstream target genes (Selvamurugan et al., 2004). BMP4 can act on tumor growth directly or indirectly by interacting with cytokines or affecting downstream gene expression. On the one hand, BMP4 promotes tumor growth by directly stimulating the expression of genes related to tumor cell proliferation and migration. For instance, in prolactin tumors, BMP4 facilitates the expression of proliferation-related genes such as C-MYC to provoke tumor cell proliferation (Paez-Pereda et al., 2003). On the other hand, BMP4 inhibits tumor growth by interacting with other cytokines. For example, in myeloma cells, BMP4 inhibits DNA synthesis depending on interleukin-6 by downregulating tyrosine phosphorylation of Stat3 induced by interleukin-6 and then inhibits tumor cell proliferation (Hjertner et al., 2001). SKP2, also known as FBXL 1, is an E3 ubiquitin ligase in the ubiquitin degradation system, forming Skp2-SCF ubiquitin ligase complex with Cullin-1, Skp1, and Ring-box 1 for degrading protein substrate. The function of SKP2 as a direct regulator of cyclin-dependent kinases and gene transcription regulators has been established (Frescas and Pagano, 2008). Accumulating studies reported that SKP2 could promote the degradation of several tumor suppressor proteins, including P27, P21, P57, and P130 and also degrade Forkhead box protein O1 by ubiquitination, a transcription factor in the FoxO family with the function of inducing cell cycle arrest, thus to promote the occurrence and development of tumors. It is detected that the protein level of SKP2 is upregulated in various types of human cancers containing liver cancer (Shin et al., 2011), lymphoma, and prostate cancer (Yang et al., 2002). Accumulating studies have reported that SKP2 has a high expression in HCC tissues, and its overexpression might indicate a poor prognosis. KNG1 gene located on chromosome three contains 11 exons expressing high molecular weight kininogene (HK) and low molecular weight kininogene (LK). KNG1 is mainly synthesized in the liver belonging to a plasma protein and a cysteine proteinase inhibitor cleaved into six subchains. KNG1 has been revealed to exert blood coagulation and important anti-inflammatory functions and inhibit the proliferation of endothelial cells and glioma cells (Xu et al., 2018). A recent study demonstrates that KNG1 is connected with the coronavirus disease 2019 infection by reducing ACE2–bradykinin axis related to lung injury and inflammation (Roche and Roche, 2020). Several studies showed that KNG1 had higher content in urine and serum from different cancers, and the content of KNG1 in serum was lower before operation than after operation in colorectal cancer patients (Navaneethan et al., 2014). Recently, KNG1 was also considered a serum biomarker for the early detection of colorectal cancer and advanced colorectal adenoma (Wang et al., 2013). It is noteworthy that the significant decrease in FASN (log2FC= −1.64), MCM5 (log2FC = −1.95), MCM6 (log2FC = −1.90), PCNA (log2FC = −2.31), MARCKS (log2FC = −2.00), FADS2 (log2FC = −3.22), CXCL1 (log2FC = −6.27), BMP4 (log2FC = −2.12), SKP2 (log2FC = −2.03), and KNG1 (log2FC = −3.12), all of which have been related to tumor cell growth, invasion, and metastasis.

Carcinogenesis is a complicated cellular process involved in the dysfunction of various biological pathways (Wang et al., 2013). Through transcriptome analysis, we determined many potential molecular pathways that are significant for carcinogenesis and tumor progression in Huh-7 cells with the treatment of metformin, such as DNA replication, cell cycle, FA biosynthesis, valine, leucine and isoleucine degradation, ECM–receptor interaction, TGF-β signaling pathway, Fanconi anemia pathway, basal cell carcinoma, synthesis and degradation of ketone bodies, microRNAs in cancer, AMPK signaling pathway, cytokine–cytokine receptor interaction, complement and coagulation cascades, TNF signaling pathway, pathways in cancer cGMP-PKG signaling pathway, and PPAR signaling pathway.

In this paper, the pathway of the cell cycle was significantly enriched in downregulated genes of Huh-7 cells treated with metformin (Supplementary Figure S1A). The cell cycle is a pathway associated with cell division mainly driven by a series of genes and other pathways. The regulation of the cell cycle is essential for cell proliferation, differentiation, and apoptosis, especially the compounds synthetized by cyclins and cyclin-dependent kinases. G1/S phase, called Restriction point (R point) in mammals, is the most important checkpoint and controls the transition of a cell from the G1 phase to the S phase. The R point mainly examines whether DNA is damaged or the extracellular environment is suitable, or the cell volume is large enough; therefore, the accurate transition of G1/S is important to the regulation of cell proliferation, and its misregulation may accelerate oncogenesis. SKP2, PCNA, and MCM families (MCM5, MCM6, and MCM7) are significant in the regulation of the G1/S transition (Bertoli et al., 2013) and were significantly downregulated in Huh-7 cells with metformin treatment. The outcomes showed that the arrest of the cell cycle induced by metformin might be a kind of mechanism that causes proliferative inhibition of Huh-7 cells.

DNA and its related substance replication are the basis of cell division, and the perfect mechanism of DNA repair in cells is beneficial to maintaining the integrity of individual genomes. DNA damage caused by intracellular or extracellular stimulation may lead to genomic instability, resulting in cell apoptosis and even malignant transformation of normal cells into tumor ones or other irreversible adverse consequences if it cannot be repaired in time. In our study, the DNA replication pathway in Huh-7 cells treated with metformin was enriched in downregulated genes such as PCNA and MCM families (MCM2, MCM4, MCM5, and MCM6) (Supplementary Figure S1B). The outcomes confirmed that the G1/S phase of cell cycle arrest caused by metformin might attribute to DNA replication.

ECM, as a main constitution of the extracellular microenvironment, plays an important role in the regulation of cell growth, metabolism, migration, proliferation, and differentiation by directly interacting with cells through integrins or other cell surface receptors and is also a significant tissue barrier for tumor metastasis (Wang et al., 2017). A study showed that the formation of ECM tumor fiber formed a cross-linked network structure, which not only helps to prop up and provide nutrition for tumor cells but also plays an important role in tumor growth and invasion. TGF-β produced by tumor cells is a kind of important regulatory factor of activating and promoting fibroblast formation and mediates tumor metastasis by promoting ECM fibrogenesis (Chakravarthy et al., 2018). The basement membrane is a significant part of ECM and acts in the process of ECM promoting tumor cell metastasis. Collagen and laminin are the main components of the basement membrane and play a crucial role in the invasion and development of cancer cells (Kim et al., 2010). In our study, the genes related to the ECM–receptor interaction pathways (Supplementary Figure S1C) are downregulated in Met-treated Huh-7 cells, such as collagen family members (COL1A1, COL1A2, COL2A1, COL9A2, COL9A3, and COL4A1), laminin family members (LAMB3, LAMC2, and LAMA3), and integrin family members (ITGA7 and ITGA5), which are highly consistent with the previous outcomes of migration and invasion assays. The outcomes demonstrated that the inhibition of migration and invasion in Huh-7 cells with metformin treatment might be worked through influencing ECM–receptor interaction pathway.

## Conclusion

In summary, this study showed that metformin had a significant inhibitory influence on the proliferation, migration, and invasion of Huh-7 *in vitro*, which was an outcome of mutual effect among a load of pathways such as DNA replication, cell cycle, ECM–receptor interaction, FA biosynthesis, AMPK signaling pathway, TNF signaling pathway, valine, leucine and isoleucine degradation, TGF-β signaling pathway, pathways in cancer, basal cell carcinoma, PPAR signaling pathway, and multiple of genes related with cell proliferation, cell adhesion, cell cycle, and microRNAs in cancer, substance metabolism, such as MCM family members and DUSP1, FASN, PCNA, SKP2, KNG1, CXCL1, FADS2, MARCKS, and BMP4. In conclusion, this work suggested that metformin might provide some therapeutic effects in the Huh-7 cell line. However, further investigation will be needed to confirm these findings in other HCC cell lines and even *in vivo* in the future study in the hope of providing a new perspective for future liver cancer therapeutic developments and developing cancer treatment drugs.

## Data Availability

The datasets presented in this study can be found in online repositories. The names of the repository/repositories and accession number(s) can be found below: https://www.ncbi.nlm.nih.gov/geo/, GSE190076.
