# Supplementary figures and images for: Transcriptome Analysis of Liver Cancer Cell Huh-7 Treated With Metformin
Source: Front Pharmacol. 2022 Mar 23;13:822023. doi: 10.3389/fphar.2022.822023 (PMC8985428; doi:10.3389/fphar.2022.822023)

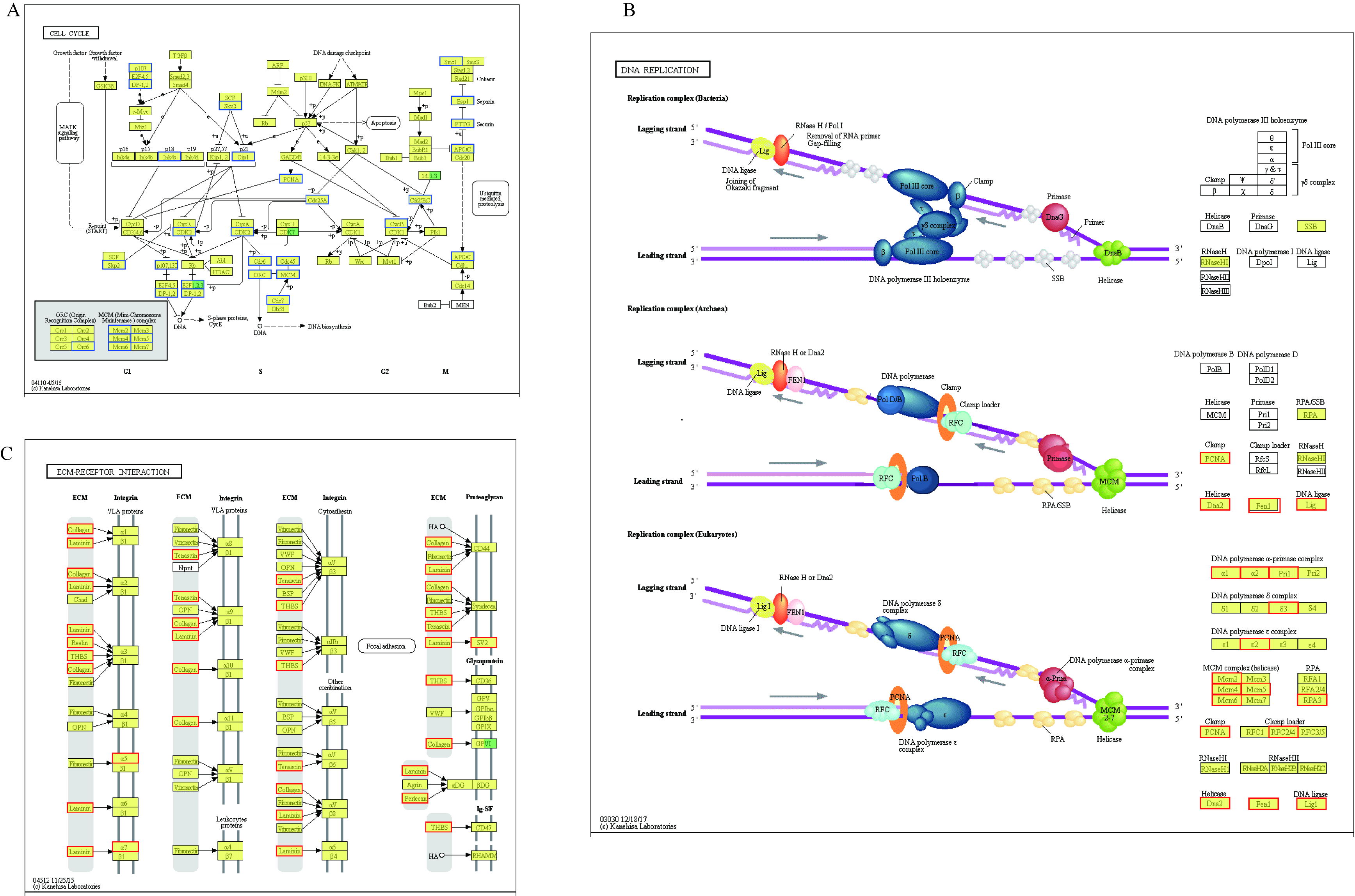

Supplement: Supplementary file 1 [file Image1.TIF]
